# Massiliamide, a cyclic tetrapeptide with potent tyrosinase inhibitory properties from the Gram-negative bacterium *Massilia albidiflava* DSM 17472^T^

**DOI:** 10.1038/s41429-020-00394-y

**Published:** 2020-12-28

**Authors:** Andri Frediansyah, Jan Straetener, Heike Brötz-Oesterhelt, Harald Gross

**Affiliations:** 1 Pharmaceutical Institute, Department of Pharmaceutical Biology, University of Tübingen, 72076 Tübingen, Germany; 2 Research Division for Natural Product Technology (BPTBA), Indonesian Institute of Sciences (LIPI), Wonosari, 55861 Indonesia; 3 Microbial Bioactive Compounds, Interfaculty Institute of Microbiology and Infection Medicine Tübingen (IMIT), University of Tübingen, Tübingen, Germany; 4 German Centre for Infection Research (DZIF), partner site Tübingen, 72076 Tübingen, Germany

**Keywords:** Natural products, Pharmaceutics

## Abstract

A cyclic tetrapeptide, designated massiliamide, was isolated from the liquid culture of the Gram-negative bacterium *Massilia albidiflava* DSM 17472^T^. The structure was elucidated through extensive spectroscopic analysis, including HR-MS and 1D and 2D NMR experiments. The absolute configuration was determined using the Marfey´s method. Massiliamide showed potent inhibitory activity towards tyrosinase with an IC_50_ value of 1.15 µM and no cytotoxicity.

*Massilia* spp. were first isolated in a human pathogenic context [1, 2] and later recognized also as air, soil, water, or plant-associated bacteria [3]. The genus *Massilia* is the most species-rich genus of the *Oxalobacteraceae* [4] and comprises currently 45 validly published species [5]. Preliminary genomic investigations indicated a significant biosynthetic potential [6]. However, despite their abundance in nature and diversity, little is known about their secondary metabolism. Natural products previously detected or isolated from *Massilia* species include indole acetic acid [7], polyhydroxyalkanoates [8], violacein [9], dimethyl disulfide [10], an agrochelin diastereomer [11], and homoserine lactones [12]. In this study, we analyzed the type strain *Massilia albidiflava* DSM 17472^T^, originally isolated from heavy metal polluted soil in Nanjing, Jiangsu Province, China [13], and present the MS-guided isolation, structure elucidation, and biological evaluation of massiliamide (**1**).

A subset of publicly available *Massilia* strains was selected (Table S1), and a liquid media screening was performed to identify new metabolites in butanol whole broth extracts by LC/MS. The screening dereplicated a variety of known metabolites, however when grown in a minimal medium, *M. albidiflava* DSM 17472^T^ produced a protonated unknown molecule at *m/z* 457 (Table S3). The cultivation was upscaled to 30 l and carried out in 15 5-l Erlenmeyer flasks, each containing 2 l of modified DMB medium (Table S2). The flasks were incubated on an orbital incubator shaker for 48 h at 30 °C and 140 rpm. Subsequently, the cells were separated from the fermentation broth by centrifugation at 4400 rpm for 30 min at 10 °C. The metabolites, secreted into the culture medium were recovered by adsorption onto Diaion HP20 resin (30 g l^−1^, shaking at 120 rpm for 4 days at 5 °C). The resin was filtered, washed twice with purified H_2_O and then, the adsorbed compounds were eluted stepwise under vacuum with solvents of decreasing polarity, ranging from a mixture of 10:90 MeOH–H_2_O to pure methanol, to give five fractions, A–E. Low-resolution LC/MS profiling indicated the third fraction to be of further interest. Separation of fraction C (76.3 mg) by RP-HPLC was performed in gradient elution mode, employing a linear gradient of 10:90 to 100:0 MeCN-H_2_O (0.1% TFA) over a period of 19 min, followed by isocratic elution at 100:0 for an additional 6 min (Phenomenex Luna Omega Polar column, 4.6 × 250 mm, 5 µm; 0.8 ml min^−1^ flow rate, UV monitoring at 254 and 280 nm), and provided massiliamide (**1**) in a semi pure form. The second round of purification was done, applying a linear gradient of 10:80 to 100:0 MeCN-H_2_O (0.1% TFA) over a period of 8 min, followed by isocratic elution at 100:0 for an additional 8 min (Phenomenex Luna C5 column, 4.6 × 250 mm, 5 μm; 0.5 ml min^−1^ flow rate; UV monitoring at 254 and 280 nm), and yielded pure **1** (*t*_R_ 10.5 min, 5.1 mg).

The physico-chemical properties of **1** are summarized as follows: Amorphous, yellow bright opaque powder; [α]${\,}^{25}_{D}$ − 20 (*c* 0.040, MeOH); UV (MeOH) λ_max_ nm (ε) 229 (4000), 293 (900); FT-IR (ATR) ν_max_ 3300, 2950, 1660, 1560, 1410, 1360 cm^-1^; ^1^H NMR and ^13^C NMR data, see Table 1; positive HR-ESIMS *m/z* 457.2443 [M + H]^+^ (calc. for C_24_H_33_N_4_O_5_ 457.2451, Δ = −1.7 ppm): The molecular formula of C_24_H_32_N_4_O_5_ was provided by HR-ESIMS and supported by NMR spectroscopic data. The IR spectrum of **1** implied the presence of OH groups, amide bonds, and an aromatic system. The ^1^H and ^13^C NMR data (Table 1) suggested that **1** was of peptidic nature due to the presence of two broad exchangeable NH singlets (*δ*_H_ 7.88, 7.98), four α-H multiplets (*δ*_H_ 3.80–4.30), several high field signals (*δ*_H_ 0.80–3.50), and four putative ester/amide carbonyl ^13^C signals (*δ*_C_ 165.2–170.3). Furthermore, in the ^1^H NMR spectrum, two mutually coupled aromatic 2H doublets (*δ*_H_ 6.63, 7.04) were observed, indicating a *para*-disubstituted benzene system. These data suggested that **1** represents a tetrapeptide, containing one aromatic and three aliphatic amino acids. A ^1^H-^13^C-HSQC-TOCSY experiment for **1** identified spin systems for one valine and two proline amino acid residues (Fig. 1). Interpretation of the ^1^H–^13^C-HSQC, selective gradient 1D-TOCSY, and ^1^H–^15^N-HSQC NMR spectra, recorded for **1**, allowed the assignment of each proton with its corresponding carbon and nitrogen resonance, respectively (Table 1). The remaining independent spin system of the type X-NH-CH-CH_2_-X´, together with ^1^H–^13^C-HMBC correlations from *β*-H_2_ (Tyr) to the γ- and *ortho*-carbons of the disubstituted benzene and the presence of two other quarternary aromatic carbons (*δ*_C_ 127.0, 155.9), thereof one oxygenated, established the tyrosine unit (Fig. 1). Consideration of the ^1^H–^13^C long-range resonances, observed between the *β*-protons to the corresponding carbonyl group completed the full shift assignment of each amino acid. However, resonances overlap between Tyr/Val carbonyls (*δ*_C_ 165.07, 165.24) and Pro/Val β-carbons (*δ*_C_ 27.66, 27.82, 27.85) were first ambiguous due to insufficient digital resolution, which was overcome by the application of band-selective HMBC experiments focused on these regions (Figs. S12–S13).

**Table 1:** ^1^H (400 MHz), ^13^C (100 MHz), and ^15^N (41 MHz) NMR spectroscopic data for massiliamide (**1**) in *d*_6_-DMSO

Unit	position	*δ*_C,N,_ mult.	*δ*_H_, mult. (*J*, Hz)
Pro1	Ν	126.0^a^	
	α	58.4, CH	4.05, dd (7.0, 7.0)
	β	27.82, CH_2_	1.39, m 2.00, m
	γ	21.8, CH_2_	1.72, m
	δ	44.5, CH_2_	3.26, m 3.41, m
	CO	168.9, qC	
Val	NH	111.8	7.98, brs
	α	59.5, CH	3.92, brs
	β	27.66, CH	2.34, m
	γ	16.4, CH_3_	0.84, d (6.9)
	γ´	18.3, CH_3_	1.01, d (7.2)
	CO	165.24, qC	
Pro2	Ν	125.6^a^	
	α	58.2, CH	4.12, m
	β	27.85, CH_2_	1.84, m 2.14, m
	γ	22.1, CH_2_	1.84, m
	δ	44.6, CH_2_	3.36, m 3.42, m
	CO	170.3, qC	
Tyr	NH	117.0	7.88, brs
	α	56.0, CH	4.24, t (4.8)
	β	34.7, CH_2_	2.92, dd (4.8, 1.0)
	γ	127.0, qC	
	*ortho*	130.8, CH	7.04, d (8.4)
	*meta*	114.7, CH	6.63, d (8.4)
	*para*	155.9, qC	
	OH		9.18, brs
	CO	165.07, qC	

^a^Interchangeable resonances

**Fig. 1:**
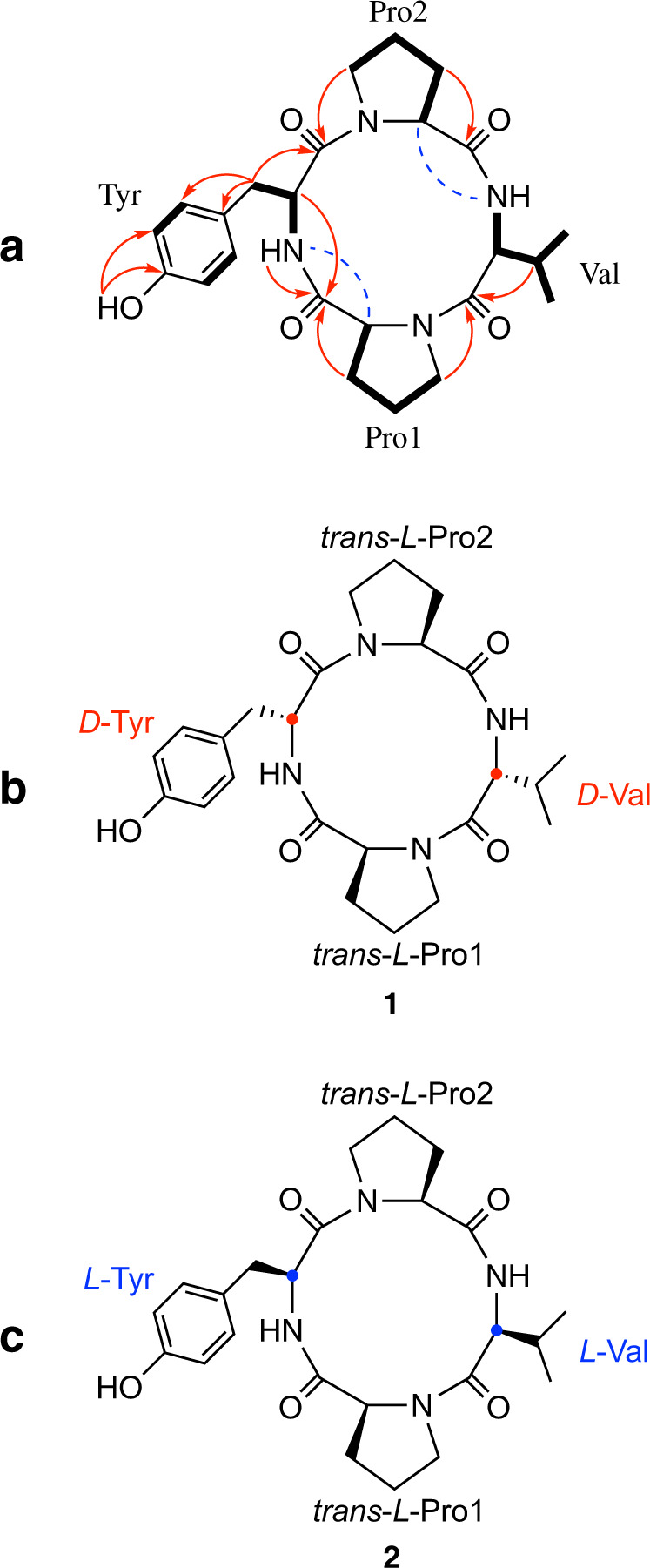
**a** Key correlations observed in ^1^H–^1^H-COSY / ^1^H–^13^C-HSQC-TOCSY (bold lines), ^1^H–^13^C-HMBC (red arrows), and ^1^H–^1^H-NOESY (dashed blue lines) NMR spectra of **1**. **b** Structure of massiliamide (**1**). **c** Structure of an unnamed *Lactobacillus*-derived cyclotetrapeptide (**2**)

In the absence of further sp^2^ carbons and having accounted for 10 of 11 elements of unsaturation, **1** was determined to be monomacrocyclic. The connectivity among the deduced amino acids was elucidated by the ^1^H–^13^C long-range couplings between *δ*-H_2_ (Pro1)/CO (Val), *δ*-H_2_ (Pro2)/CO (Tyr), *α*-H (Tyr)/CO (Pro1), NH (Tyr)/CO (Pro1) along with NOE correlations of NH (Tyr)/*α*-H (Pro1), and NH (Val)/*α*-H (Pro2) (Fig. 1) established the amino acid sequence.

The absolute configuration of the amino acids was clarified by Marfey´s method [14] applied to the acid hydrolysate of **1** in comparison with standard amino acids (Fig. S17) and was found to be 2 × L-Pro, 1 × D-Tyr, and 1 × D-Val (Fig. 1). Since amides attached through a proline nitrogen are known to be capable of existing as equilibrating rotamers, the geometry of the proline peptide bonds required resolution. According to empirical rules, ^13^C NMR chemical shift differences between proline *β* and *γ* carbon resonances are characteristic of *cis* (Δ*βγ* ∼8–12 ppm) vs. *trans* (Δ*βγ* ∼2–6 ppm) rotamers, respectively [15]. Thus, the ^13^C NMR data indicated that both proline peptide bonds were *trans* configurated, as shown by the small chemical shift differences of Pro1 Δ*βγ* = 6.0 and Pro2 Δ*βγ* = 5.8, respectively (Table 1).

According to a literature search, **1** possesses the same planar structure as an unnamed cyclotetrapeptide (**2**) which was isolated from the Gram-positive bacterium *Lactobacillus helveticus* JCM 1120 [16]. However, the two compounds differed in the absolute configuration of the involved amino acids (Pro1-Tyr-Pro2-Val). While **1** displays a L/D/L/D-configuration, the *Lactobacillus*-tetrapeptide (**2**) represents the corresponding *all-*L-version, also with both proline peptide bonds in *trans* configuration (Fig. 1). Thus, **1** is a new stereoisomer of **2** and we therefore suggest the trivial name massiliamide for **1**.

In standardized antimicrobial assays by broth microdilutions, **1** was inactive up to the highest concentration tested (64 µg ml^−1^) and showed no cytotoxicity towards the HeLa cell line at 64 µg ml^−1^ (Table S4). However, in a tyrosinase inhibition assay, **1** proved with an IC_50_ value of 1.15 µM to be a potent tyrosinase inhibitor which surpassed readily the activities of the positive controls arbutin and kojic acid (Table S5, Figs. S18 and S19).

In summary, we isolated a new tetrapeptide with potent tyrosinase inhibitory activity, which we designated massiliamide (**1**). Considering the chemical space [17–19] of the so far known tyrosinase inhibitors, **1** represents a member of the rather rare class of natural peptide-based tyrosinase inhibitors [17, 20]. The enzyme tyrosinase is involved in melanogenesis and determines the color of skin and hair. Furthermore, unfavorable enzymatic browning of plant-derived food products is also mediated by tyrosinase and causes a significant economic loss. Thus, nontoxic and potent inhibitors such as massiliamide may find application in cosmetics and medical industry as depigmentation agents as well as in food and agricultural industries as antibrowning compounds.

## Supplementary information

SUPPLEMENTAL MATERIAL
