# Supplementary material for: Massiliamide, a cyclic tetrapeptide with potent tyrosinase inhibitory properties from the Gram-negative bacterium Massilia albidiflava DSM 17472T
Source: J Antibiot (Tokyo). 2020 Dec 28;74(4):269–72. doi: 10.1038/s41429-020-00394-y (PMC8005375; doi:10.1038/s41429-020-00394-y)
Supplement: Supplementary file 1 — SUPPLEMENTAL MATERIAL [file 41429_2020_394_MOESM1_ESM.docx]

**Massiliamide, a cyclic tetrapeptide with potent tyrosinase inhibitory properties from the Gram-negative bacterium *Massilia albidiflava* DSM 17472^T^**

**Andri Frediansyah, Jan Straetener, Heike Brötz-Oesterhelt, Harald Gross**

**SUPPLEMENTAL INFORMATION**

**Table of Contents**

**General experimental procedures**

**Screening of *Massilia* spp. strains**

Table S1 *Massilia* spp. strains used for screening and production in this study

Table S2 Media composition

MS-based screening

Table S3 LC/MS screening results of the investigated strains

**Spectral Data for Massiliamide (1)**

Figure S1 UV spectrum

Figure S2 FT-IR spectrum

Figure S3 HR-ESI-MS spectrum

Figure S4 400 MHz ^1^H NMR spectrum of **1** in *d*_6_-DMSO.

Figure S5 100 MHz ^13^C NMR spectrum of **1** in *d*_6_-DMSO.

Figure S6 400 MHz DEPT135 NMR spectrum of **1** in *d*_6_-DMSO.

Figure S7 400 MHz multiplicity edited ^1^H-^13^C-HSQC NMR spectrum of **1** in *d*_6_-DMSO.

Figure S8 400 MHz selective gradient TOCSY of **1** in *d*_6_-DMSO.

Figure S9 400 MHz ^1^H-^1^H-COSY NMR spectrum of **1** in *d*_6_-DMSO.

Figure S10 400 MHz HSQC-TOCSY spectrum of **1** in *d*_6_-DMSO.

Figure S11 400 MHz ^1^H-^13^C-HMBC NMR spectrum of **1** in *d*_6_-DMSO.

Figure S12 400 MHz band-selective ^1^H-^13^C-HMBC NMR spectrum of **1** (*δ*_C_ 25-36).

Figure S13 400 MHz band-selective ^1^H-^13^C-HMBC NMR spectrum of **1** (*δ*_C_ 150-176).

Figure S14 400 MHz ^1^H-^1^H-NOESY NMR spectrum of **1** in *d*_6_-DMSO.

Figure S15 400 MHz ^1^H-^15^N-HSQC NMR spectrum of **1** in *d*_6_-DMSO.

Figure S16 400 MHz ^1^H-^15^N-HMBC NMR spectrum of **1** in *d*_6_-DMSO.

**Determination of the Absolute Configuration of Massiliamide (1)**

Figure S17 LC/MS analysis of the peptide hydrolysate of **1**.

**Biological Assays**

Table S4 Results of the antimicrobial and cytotoxicity assays

Table S5 Results of the tyrosinase inhibition assay

Figure S18 Concentration-dependent inhibitory effects of **1**

Figure S19 IC_50_-determination

**Supplemental References**

**General experimental procedures**

HPLC was performed with a Waters system, controlled by the Waters Millenium Software 4.0 and consisting of a Waters 1525 pump, a Waters 996 photodiode array detector, a Rheodyne 7725i injector and a Kromega vacuum degasser series A50010. For LC-MS analysis, an 1100 Series HPLC system was fitted with a G1322A degasser, a G1312A binary pump, a G1329A autosampler, and a G1315A diode array detector. The Agilent HPLC components were connected with an ABSCIEX 3200 QTRAP LC/MS/MS mass spectrometer. 1D and 2D NMR spectra were measured on a Bruker Avance III HD 400 spectrometer using either a 5 mm SMART probe head or a 3 mm inverse probe head. All spectra were recorded in *d*_6_-DMSO at 298 K and were referenced to the residual hydrogenated solvent signals with resonances at *d*_H/C_ 2.50/39.5 or to the internal offset for ^15^N assigned by the instrument manufacturer (Bruker), respectively. The obtained NMR raw data were processed and analyzed using MestReNova 14.1.2, applying standard parameters except for the processing of band-selective 2D ^1^H-^13^C-HMBC experiments. In the latter case, upon a regular 2D Fourier transformation, band-selective HMBC data sets were further processed by calculation of the magnitude spectrum in the F2 dimension and the contour display graphically optimized concerning the ´scaling´ (1.5 instead of 2) and the ´line width´ (5 instead of 2.5) parameters. The optical rotation value was measured on a Jasco P-2000 polarimeter, using a 3.5 mm x 10 mm cylindrical quartz cell. Infrared spectra were obtained employing a Jasco FT/IR 4200 spectrometer, interfaced with a MIRacle ATR device (ZnSe crystal). High-resolution mass spectra were acquired on an HR-ESI-TOF-MS Bruker maXis 4G mass spectrometer. All solvents were purchased as HPLC or LC-MS grade, respectively.

**Screening of *Massilia* spp. strains**

The type strain *Massilia timonae* LMG 21530^T^ was purchased from the BCCM/LMG – Belgian Coordinated Collections of Microorganisms / Bacteria Collection Laboratorium voor Microbiologie Universiteit Gent, Ghent, Belgium. All other *Massilia* type strains (Table S1) were obtained from the DSMZ – German Collection of Microorganisms and Cell Cultures GmbH, Braunschweig, Germany and treated according to the instructions given.

**Table S1.** *Massilia* spp. strains used for screening and production in this study

| **Organism** | **Strain No.** | **Bio-Safety-Level^a^** | **Reference** |
| --- | --- | --- | --- |
| *Massilia albidiflava* | DSM 17472^T^ | 1 | [1] |
| *Massilia dura* | DSM 17513^T^ | 1 | [1] |
| *Massilia flava* | DSM 26639^T^ | 1 | [2] |
| *Massilia lutea* | DSM 17473^T^ | 1 | [1] |
| *Massilia plicata* | DSM 17505^T^ | 1 | [1] |
| *Massilia umbonata* | DSM 26121^T^ | 1 | [3] |
| *Massilia timonae* | LMG 21530^T^ | 1 | [4,5] |

^a^according to the classification of the corresponding culture collections

**Table S2.** Media composition.

| **Medium** | **Ingredients and composition** |
| --- | --- |
| Luria-Bertani (LB): | 10 g tryptone, 5 g yeast extract, 10 g NaCl, final pH 7.5±2, at 25^o^C |
| Reasoner’s 2 A (R2A): | 0.5 g yeast extract, 0.5 g protease peptone, 0.5 g casamino acids, 0.5 g dextrose, 0.5 g soluble starch, 0.3 g sodium pyruvate, 0.3 g KH_2_PO_4_, 50 mg MgSO_4_ x 7H_2_O, final pH 7.2±2 at 25^o^C |
| Modified Davis Minimal Broth DMB: | 5.3 g Davis minimal broth without dextrose, 0.56 g HEPES buffer, 5.1 g sucrose, 1 mg methionine, 1 ml A5+co trace metal mix, final pH 6.6±2 at 25^o^C  A5+co trace metal mix: 2.86 g H_3_BO_3_, 1.81 g MnCl_2_ x 4H_2_O, 0.222 g ZnSO_4_ x 7H_2_O, 0.390 mg Na_2_MoO_4_ x 2H_2_O, 79 mg CuSO_4_ x 5H_2_O, 49 mg Co (NO_3_)_2_ x 6H_2_O, pH adjustment is not necessary |
| Shipworm basal medium (SBM) without sea salt:  (modified from [6]) | 15.3 mg KH_2_PO_4_, 10 mg Na_2_CO_3_, 2.5 mg Na_2_MoO_4_ x 2H_2_O, 0.5 mg EDTA, 3 mg C_6_H_8_FeNO_7_, 5.2 g HEPES buffer, 1mL A5-co trace metal mix, pH adjustment is not necessary |
| MM9:  Reference [7] | 900 ml Solution C, 20 ml Solution A, 20 ml Solution B, 16.7 ml 100mM L-leucine, 5 ml 60mM L-histidine, 10 ml 100 mM L-lysine, 10 ml 40 mM L-tryptophan, 10 ml 40 mM L-methionine, 20 ml 50% (w/v) glucose, 1 mL A5-co trace metal mix, pH adjustment is not necessary  Solution A: 100 g KH_2_PO_4,_ 350 g K_2_HPO_4_, 0.20 g, pH adjustment is not necessary  Solution B: 50 g (NH_4_)_2_SO_4_, 5 g MgSO_4,_ pH adjustment is not necessary  Solution C: 2 g of each amino acid (L-arginine, L-alanine, L-asparagine, L-aspartate, L-cysteine, L-glutamine, L-glutamate, L-glycine, L-isoleucine, L-proline, L-serine, L-threonine, L-tyrosine, L-valine, and L-phenylalanine), final pH 7.2±2 at 25^o^C |
| SRM_HG_:  Reference [8] | 4 g L-histidine, 1.6 mg FeCl_3_, 0.89 g KH_2_PO_4,_ 0.8 g K_2_HPO_4_, 0.20 g, MgSO_4_ x 7 H_2_O, 10 g glucose, 1 g fructose, pH adjustment is not necessary |
| Modified SRM_HG_: | 4 g L-histidine, 1.6 mg FeCl_3_, 0.89 g KH_2_PO_4,_ 0.8 g K_2_HPO_4_, 0.20 g, MgSO_4_ x 7 H_2_O, 10 g glucose, 1 g fructose, 3 mg ibuprofen, 5 g casamino acids, final pH 7.2±2 at 25^o^C |

**MS-based screening for secondary metabolites from 7 *Massilia* strains using different cultivation conditions**

A volume of 200 µL of a defrosted cryo stock of each *Massilia* type strain was used to establish first a well-grown colony on solid lysogeny broth (LB) medium (Table S2) within 48 h at 30°C. Subsequently, a 15 mL cell culture centrifuge tube containing 6 mL of LB liquid medium was inoculated with picked colonies from the solid preculture, and incubated at 30°C and 250 rpm for 24 h, using a VWR orbital shaker model 3500, to produce a seed culture. Seven further precultures of each strain were then prepared by dilution to an OD_600_ of 0.06 into 14 new 15 mL sterile cell culture centrifuge tubes containing each 6 mL LB medium and which were re-incubated under the same conditions. Two 5 L-Erlenmeyer flasks containing each 2.5 L of either SBM, modified DMB, MM9, SRM_HG_, modified SRM_HG_, LB or R2A medium (Table S2), respectively, were inoculated with 6 mL starter culture. The cultures were incubated at 30°C with (140 rpm) and without shaking for 48 h. Notably, the shaking was essential, since fermentations without shaking caused only marginal growth. Thus, all experiments without shaking were not further investigated. The culture broth of each fermentation was then extracted separately with butanol at a ratio of 1:1, and evaporated under reduced pressure. In order to reduce the sample complexity and to minimize the suppression of ionization by high abundance species, each crude extract was fractionated, employing reversed-phase (RP) vacuum liquid chromatography (VLC), prior to LC/MS analysis. The RP C_18_ column (employing a 300 mL fritted-funnel, Macherey-Nagel Polygoprep 60-50 C_18_, 11.1 cm x 7.9 cm) was eluted stepwise under vacuum with solvents of decreasing polarity, ranging from a 10:90 MeOH/H_2_O mixture to pure MeOH to give five fractions. The resultant fractions of each experiment were dissolved in LC/MS grade methanol and profiled by LC/MS using a MeOH/H_2_O (0.1% TFA) gradient, increasing the MeOH portion from 10 to 30% over 8 min and from 30 to 100% over 22 min (Phenomenex Luna C18(2) column, 4.6 × 250 mm, 3 μm; 0.2 ml min^−1^ flow rate; with total ion current [positive mode] and photodiode array monitoring).

**Table S3.** LC/MS screening results of the investigated strains.

| **Bacterial strains** | **Medium** | | | | | | |
| --- | --- | --- | --- | --- | --- | --- | --- |
|  | **SBM without sea salt** | **Modified DMB I** | **MM9** | **SRM_HG_** | **Modified SRM_HG_** | **LB** | **R2A** |
| *Massilia lutea* | 537.12 (β-carotenoid),  364.9, 670.3 | - | - | 537.12 (β-carotenoid) | 537.12 (β-carotenoid) | 537.12 (β-carotenoid) | - |
| *M. dura* | - | - | - | - | - | - | - |
| *M. albidiflava* | 537.12 (β-carotenoid) | 537.12 (β-carotenoid),  457.27 ^a^ (unknown) | 748.1 (Rhizomide B) | - | 537.12 (β-carotenoid) | - | 537.12 (β-carotenoid) |
| *M. umbonata* | 537.12 (β-carotenoid),  364.9, 670.3 | - | - | - | - | - | 537.12 (β-carotenoid) |
| *M. flava* | 36.9 | 537.12 (β-carotenoid) | 732.19  (Rhizomide A),  537.12 (β-carotenoid) |  | 732.19 (Rhizomide A),  553.30  (epoxy-carotene suspected) | - | 537.12 (β-carotenoid) |
| *M. plicata* | - | - | - | - | - | - | - |
| *M. timonae* | - | - | - | - | - | - | - |
|  |  |  |  |  |  |  |  |

**^a^**The screening experiment was repeated, but this time, the supernatant and cells were analyzed separately.

This procedure indicated that the target compound (*m/z* 457) is predominantly present in the supernatant.

**Spectral Data for Massiliamide (1)**

**Figure S1.** UV spectrum of **1** in MeOH.


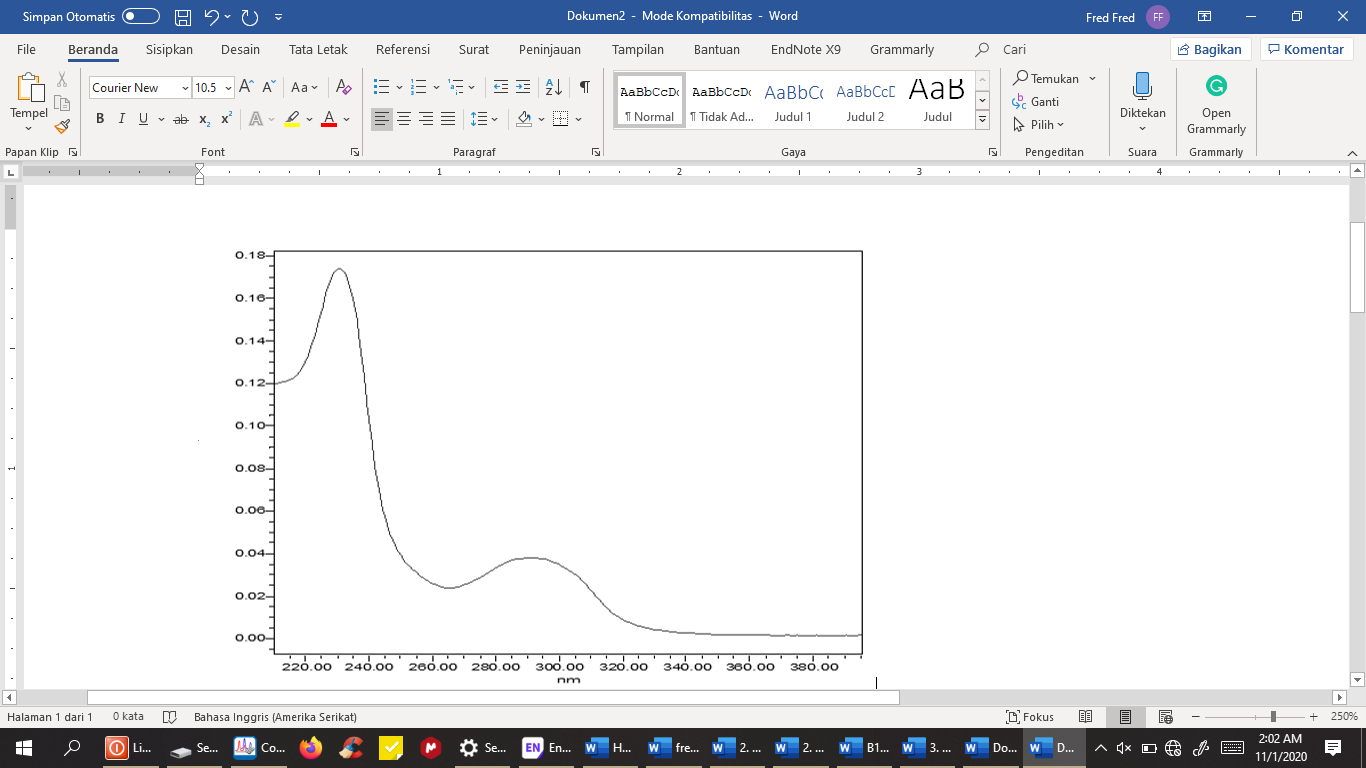


**Figure S2.** FT-IR of **1**.


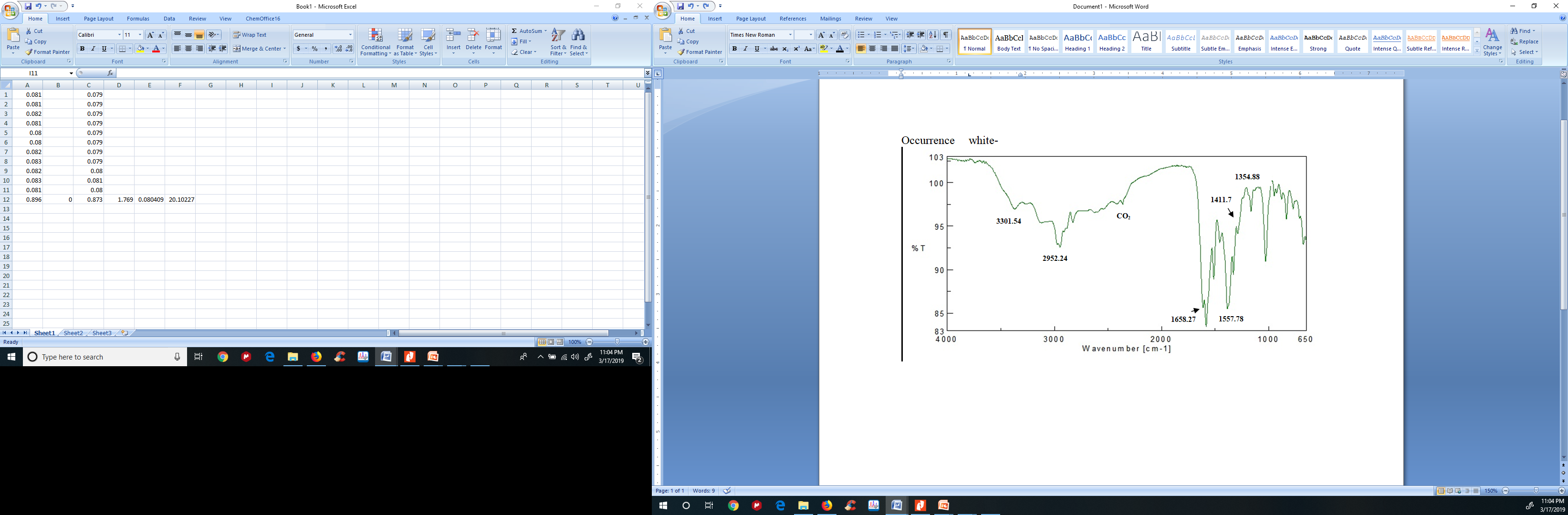


**Figure S3.** HR-ESI-MS spectrum of **1**.


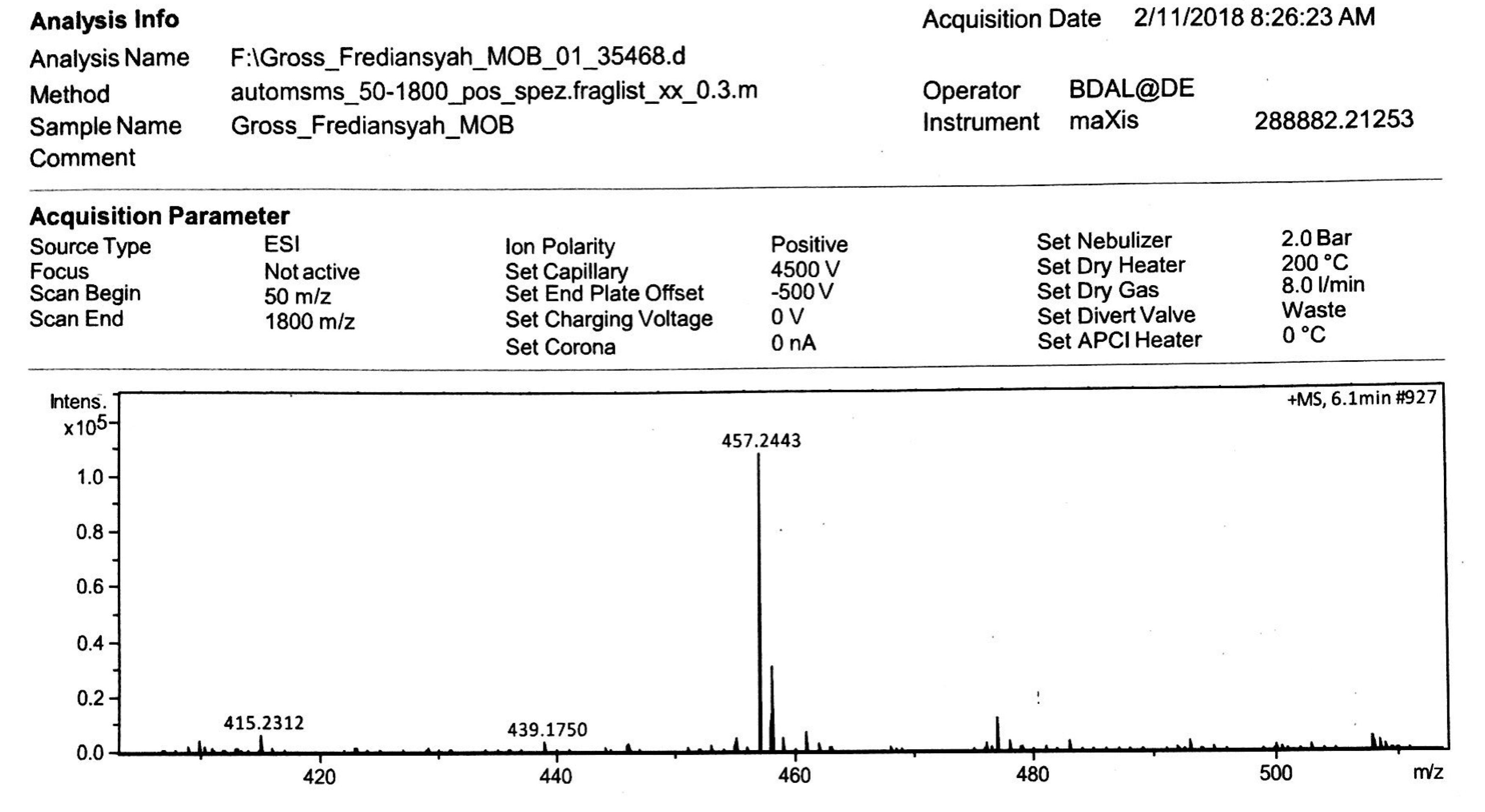


positive HRESIMS *m/z* 457.2443 [M+H]^+^ (calc. for C_24_H_33_N_4_O_5_ 457.2451, $\Delta$ = -1.7 ppm)

**Figure S4.** 400 MHz ^1^H NMR spectrum of **1** in *d*_6_-DMSO.

**Figure S5.** 100 MHz ^13^C NMR spectrum of **1** in *d*_6_-DMSO.

**Figure S6.** 400 MHz DEPT135 NMR spectrum of **1** in *d*_6_-DMSO.

**Figure S7.** 400 MHz multiplicity edited ^1^H-^13^C-HSQC NMR spectrum of **1** in *d*_6_-DMSO.

**Figure S8.** 400 MHz selective gradient 1D-TOCSY NMR spectrum of **1** in *d*_6_-DMSO.

Irradiation of ***β*-H (Pro1)** at *δ*_H_ 1.39.

*α*−H (Pro1)

*δ*_H_ 4.05

*δ*−H_2_ (Pro1)

*δ*_H_ 3.26

*δ*_H_ 3.41

*β*−H_2_ (Pro1)

*δ*_H_ 1.39

*δ*_H_ 1.72

*γ*−H_2_ (Pro1)

*δ*_H_ 1.72

**Figure S9.** 400 MHz ^1^H-^1^H-COSY NMR spectrum of **1** in *d*_6_-DMSO.

**Figure S10.** 400 MHz HSQC-TOCSY NMR spectrum of **1** in *d*_6_-DMSO.

**Figure S11.** 400 MHz ^1^H-^13^C-HMBC NMR spectrum of **1** in *d*_6_-DMSO.

**Figure S12.** 400 MHz band-selective ^1^H-^13^C-HMBC NMR spectrum of **1** (*δ*_C_ 25-36).

***β*-C (Pro1)** ***δ*_C_ 27.82**

***β*-C (Pro2) *δ*_C_ 27.85**

***α*-H (Pro2) *δ*-H (Pro2)**

***δ*_H_ 4.12 *δ*_H_ 3.36**

***α*-H (Pro1) *δ*-H (Pro1)**

***δ*_H_ 4.05 *δ*_H_ 3.26**

**Figure S13.** 400 MHz band-selective ^1^H-^13^C-HMBC NMR spectrum of **1** in *d*_6_-DMSO

(focused on *δ*_C_ 150-176).

**C=O (Tyr) *δ*_C_ 165.1**

**C=O (Val) *δ*_C_ 165.2**

***α*-H (Val) *β*-H_2_ (Tyr)**

***α*-H (Tyr) *δ*_H_ 3.92  *δ*_H_ 2.92  *β*-H(Val)**

***δ*_H_ 4.24 *δ*_H_ 2.34**

**Figure S14.** 400 MHz ^1^H-^1^H-NOESY NMR spectrum of **1** in *d*_6_-DMSO.

**Figure S15.** 400 MHz ^1^H-^15^N-HSQC NMR spectrum of **1** in *d*_6_-DMSO.

**Figure S16.** 400 MHz ^1^H-^15^N-HMBC NMR spectrum of **1** in *d*_6_-DMSO.

**Determination of the Absolute Configuration of Massiliamide (1)**

1 mg of massiliamide was dissolved in 2000 µL of 6N DCl/D_2_O (deuterated hydrochloric acid) and hydrolysis allowed to proceed at 110 °C for 24 h according to the method of Kusumoto *et al*. [9] in order to detect misleading racemization. The solvent was then evaporated in vacuo and the remaining hydrolysis residue was re-dissolved in water (100 µL). 200 µL of 1% Marfey’s reagent in acetone (1-fluoro-2,4-dinitrophenyl-5-L-alanine amide, FDAA) and 40 *µ*L 1.0 M sodium bicarbonate solution were added. The reaction mixture was then heated with shaking for 1 h at 40°C. Subsequently, the solution was cooled down to room temperature and the reaction was stopped by the addition of 20 µL of 2N HCl and centrifuged for 10 min at 3000 rpm. The derivatized amino acids were analyzed by LC/MS. The LC/MS was equipped with a Phenomenex Luna C18(2) column (4.6 x 250 mm, 3 µm). A linear gradient of 10:90 to 100:0 MeCN-H_2_O (0.1% TFA) over a period of 5 min was employed, followed by isocratic elution at 100:0 for an additional 25 min (0.2 mL min^-1^ flow rate, UV monitoring at 340 nm). For MS detection, the following parameters were optimized: curtain gas 25 psi, temperature 450°C, gas 1 and 2: 30 psi, ion spray voltage -4500 V, declustering potential 46 V, entrance potential 10 V, and interface heater on. L- and D- amino acid standards for the assignment of the absolute configuration were dissolved in 100 µl H_2_O and treated in the same manner using FDAA. Each of the peaks in the chromatographic trace was identified by comparing its retention time (L-Pro, D-Pro, L-Val, D-Val, L-Tyr, and D-Tyr was 29.58, 30.15, 32.42, 33.72, 34.65, and 35.48, respectively) and mass spectrum with that of the corresponding amino acid standard (Figure S16) and by co-injection.

**Figure S17.** LC/MS analysis of the amino acid standards (top) and of the peptide hydrolysate of **1** (bottom). Abbreviations: LP = L-Proline, DP = D-Proline, LV = L-Valine, DV = D-Valine, LT = L-Tyrosine, DT = D-Tyrosine. The colors indicate the extracted ion of the MS trace [M+H]^+^: *m/z* 368.1, 370.1, and 434.1 for L/D-Pro, L/D-Val, and L/D-Tyr, respectively.


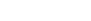

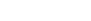

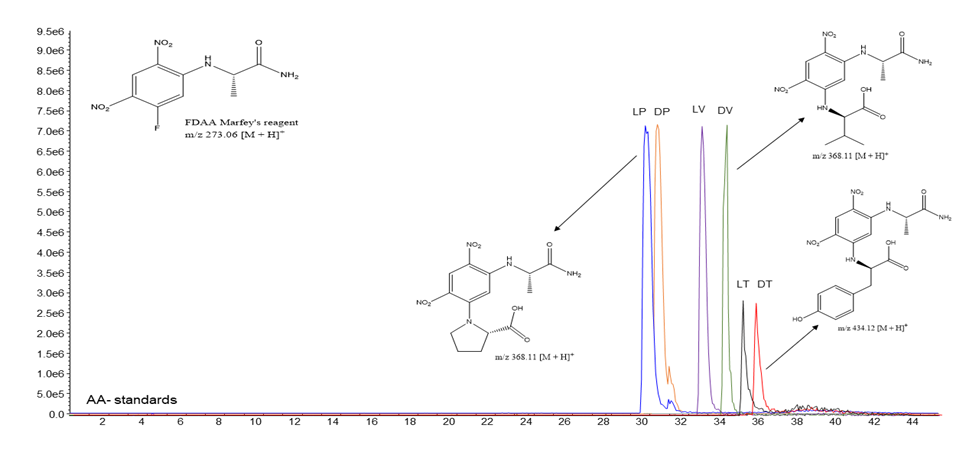


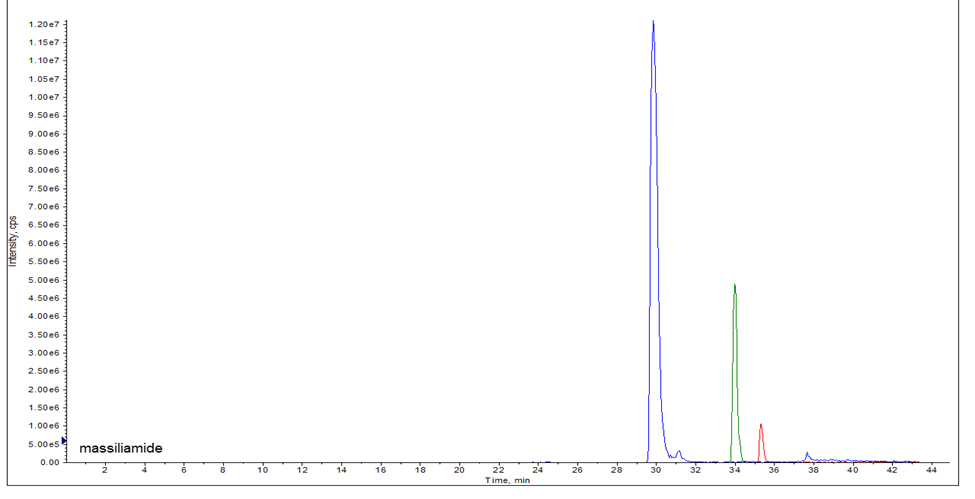


**Bioassays**

**Antibacterial assays**

The minimal inhibitory concentration (MIC) was determined in a cation-adjusted Mueller-Hinton medium that contains casein, beef extract and starch by using a twofold serial dilution method according to the standards and guidelines of the Clinical and Laboratory Standards Institute (CLSI) [10]. In brief, a twofold serial dilution of the test compound was prepared in microtiter plates and seeded using a final inoculum of bacteria of 5 × 10^5^ colony-forming units per mL. After overnight incubation at 37°C in ambient air, the MIC was determined as the lowest compound concentration preventing visible bacterial growth. The strain panel included representative species of nosocomial pathogens, which are known as “ESKAPE” bacteria. Specifically, the following strains were used: *Enterococcus faecium* BM 4147–1, *Staphylococcus aureus* ATCC 29213, *Klebsiella pneumoniae* ATCC 12657, *Acinetobacter baumannii* 09987, *Pseudomonas aeruginosa* ATCC 27853 and *Enterobacter aerogenes* ATCC 13048. *Bacillus subtilis* 168 and *Escherichia coli* ATCC 25922 were used as further reference strains. The ATCC strains were provided by the American Type Culture Collection. *A. baumannii* 09987 was obtained from the University of Bonn, Germany.

**Cytotoxicity assay**

The cytotoxicity test against the HeLa human cervical carcinoma cell line was performed in RPMI cell culture medium supplemented with 10% fetal bovine serum using the 7-hydroxy-3*H*-phenoxazin-3-one-10-oxide (resazurin) assay. A twofold serial dilution of the test compounds was prepared in duplicates in a microtiter plate and seeded with trypsinized HeLa cells to a final cell concentration of 1 × 10^4^ cells per well. After 24 h incubation at 37°C, 5 % CO_2_, 95% relative humidity, resazurin was added at a final concentration of 200 µM, and cells were again incubated overnight. Cell viability was assessed by determining the reduction of resazurin to the fluorescent resorufin. Fluorescence was measured in a TECAN M200 reader at an excitation wavelength of 560 nm and an emission wavelength of 600 nm in relation to the untreated control.

**Table S4.** Results of the antimicrobial and cytotoxicity assays

| **Bioassay** | **Massiliamide (1)** |
| --- | --- |
|  |  |
|  |  |
| **Antimicrobial activity** | **MIC (μg/ml)** |
| *E. faecium* BM 4147-1 | > 64 |
| *S. aureus* ATCC 2921 | > 64 |
| *K. pneumonia* ATCC 12657 | > 64 |
| *A. baumannii* 09987 | > 64 |
| *P. aeruginosa* ATCC 27853 | > 64 |
| *E. aerogenes* ATCC 13048 | > 64 |
| *E. coli* ATCC 25922 | > 64 |
| *B. subtilis* 168 | > 64 |
|  |  |
|  |  |
| **Cytotoxicity** | **IC_50_ (μg/ml)** |
| HeLa cell line | > 64 |

**Tyrosinase inhibition assay**

The tyrosinase activity was examined using the tyrosinase inhibitor screening kit MAK257, purchased from Sigma Aldrich and its protocol. In brief, 20 µL massiliamide (**1**) with various concentrations (0.05–5 µM) was added into 50 µL tyrosinase enzyme solutions that contained 48 µL MAK257A buffer and 2 µL of tyrosinase. The mixture was then incubated for 10 min at 25°C, followed by the addition of 30 µL substrate solution, containing 23 µL MAK257A buffer, 2 µL tyrosinase substrate, and 5 µL tyrosinase enhancer. The final reaction mixture was then homogenized by shaking and immediately monitored at 510 nm using a Tecan Infinite 200 Pro microplate reader, applying the kinetic mode for 30-60 min. Kojic acid and arbutin were used separately as positive controls. Each measurement was conducted in three independent replications. The result was determined as % relative inhibition as follows: % Relative = [Slope(EC)–Slope(S)]/Slope(EC) x 100 inhibition (EC: means enzyme activity control; S = test sample) The IC_50_ value, the concentration with 50 % inhibition of tyrosinase activity, was determined by interpolation of dose-response curves from log(inhibitor) vs. normalized relative inhibition using GraphPad Prism 7.0.

**Table S5.** Results of the tyrosinase inhibition assay

| **Bioassay** | **Massiliamide (1)** | **Arbutin** | **Kojic acid** |
| --- | --- | --- | --- |
|  |  |  |  |
| **Tyrosinase Inhibition** | **IC_50_ (μM)** | **IC_50_ (μM)** | **IC_50_ (μM)** |
|  | 1.15 ± 1.04 | 4.22 ± 1.02 | 37.09 ± 1.01 |
|  |  |  |  |

**Figure S18.** Concentration-dependent inhibitory effects of test compound **1**, arbutin and kojic acid on the activity of tyrosinase. The error bar indicates the standard error of the mean (SEM) of three independent experiments.

**Figure S19.** IC_50_ of tyrosinase inhibitory activity by massiliamide (**1**). Positive controls were included using arbutin and kojic acid.

|  |  |
| --- | --- |
|  |  |

**Supplemental References**

(1) Zhang YQ et al. Int J Syst Evol Microbiol. 2006; 56: 459-463.

(2) Wang J et al. Int J Syst Evol Microbiol. 2012; 62: 580-585.

(3) Rodríguez-Díaz M et al. Int J Syst Evol Microbiol. 2014; 64: 131-137.

(4) La Scola B, Birtles RJ, Mallet MN, Raoult D. J Clin Microbiol. 1998; 36: 2847-2852.

(5) Lindquist D et al. J Clin Microbiol. 2003; 41: 192-196.

(6) Han AW et al. PLoS ONE 2013; 8: e76151.

(7) Hermenau R et al. Nature Chem Biol. 2018; 14: 841-843.

(8) Jiao J, Du J, Frediansyah A, Jahanshah G, Gross H. J Antibiot. 2020; 73: 28-34.

(9) Kusumoto S, Matsukura M, Shiba T. Biopolymers 1981; 20: 1869–1875.

(10) National Committee for Clinical Laboratory Standards. Methods for Dilution Anti-microbial Susceptibility Tests for Bacteria that Grow Aerobically. Approved Standards. NCCLS Document M7-A4 4th ed. 1997.
